# Salt Adaptation and Evolutionary Implication of a *Nah*-related PAHs Dioxygenase cloned from a Halophilic Phenanthrene Degrading Consortium

**DOI:** 10.1038/s41598-017-12979-z

**Published:** 2017-10-02

**Authors:** Chongyang Wang, Guang Guo, Yong Huang, Han Hao, Hui Wang

**Affiliations:** 1 State Key Joint Laboratory of Environment Simulation and Pollution Control, School of Environment, Tsinghua University, Beijing, 100084 China; 2 Present Address: School of Environmental Engineering, Nanjing Institute of Technology, Nanjing, 211167 China

## Abstract

Polycyclic aromatic hydrocarbons (PAHs) pollutions often occur in marine and other saline environment, largely due to anthropogenic activities. However, study of the PAHs-degradation genotypes in halophiles is limited, compared with the mesophilic terrestrial PAHs degraders. In this study, a bacterial consortium (CY-1) was enriched from saline soil contaminated with crude oil using phenanthrene as the sole carbon source at 10% salinity. CY-1 was dominated by the moderate halophilic *Marinobacter* species, and its dominant PAHs ring-hydroxylating dioxygenase (RHD) genotypes shared high identity to the classic *nah*-related RHDs found in the mesophilic species. Further cloning of a 5.6-kb gene cluster from CY-1 unveiled the existence of a new type of PAHs degradation gene cluster (*hpah*), which most probably evolves from the *nah*-related gene clusters. Expression of the RHD in this gene cluster in *E. coli* lead to the discovery of its prominent salt-tolerant properties compared with two RHDs from mesophiles. As a common structural feature shared by all halophilic and halotolerant enzymes, higher abundance of acidic amino acids was also found on the surface of this RHD than its closest *nah*-related alleles. These results suggest evolution towards saline adaptation occurred after horizontal transfer of this *hpah* gene cluster into the halophiles.

## Introduction

Polycyclic aromatic hydrocarbons (PAHs) are a group of pollutants that consist of two or more fused benzene rings and are prevailing in both terrestrial and marine environment mainly due to anthropogenic activities^1,2^. Microbial biodegradation is the main process for elimination of PAHs in the environment^1,3^. In bacteria, PAHs degradation is initialized via di-hydroxylation catalyzed by ring-hydroxylating dioxygenase (RHD)^3^, which is composed of four components, namely a large subunit (coded by *pahAc*), a small subunit (*pahAd*), a reductase (*pahAa*), and a ferredoxin (*pahAb*)^4,5^. RHD, the large subunit in particular, is often used as a marker to detect PAHs degraders^6–8^, and the phylogenies of RHD can reflect genotypes of PAHs degraders. So far, more than ten types of gene clusters (*pah*) that consist of PAHs degradation genes have been identified^9–15^, and most of them are from terrestrial PAHs degraders. *Pah* gene clusters of the same type are often found within the same genus, family, or order^16–18^.

Although PAHs pollutions occur frequently across marine and other saline environments, genotypes of PAHs degraders from saline environment are poorly characterized. So far, marine PAHs degraders within dozens of genera have been isolated^1^; however, only two genotypes with the archetype in *Cycloclasticus* sp. A5^10^ and *Alteromonas* sp. SN2^13^ have been reported. While strain A5′s the genotype was exclusively found within *Cycloclasticus* ^19,20^, strain SN2’s genotype was found across *Alteromonas*, *Neptunomonas* ^21^, and *Pseudoalteromonas* ^22^ genera. Both of these genotypes are distinct from their counterparts in terrestrial PAHs degraders. Indeed, this segregation of genotype is common for all terrestrial microorganisms and marine microorganisms, which was mainly caused by the molecular mechanisms to adapt the distinct salt concentrations^23^. Therefore, novel PAH-degrading genotypes are expected to be existed in saline environments and their molecular mechanisms to salt adaption remain to be investigated.

Noticeably, a PAHs-degrading *Marinobacter* strain (NCE312) was once reported to have a *pahAc* fragment similar to the dioxygenase of *nah*-related genotypes^24^. *Nah*-related gene clusters are a set of evolutionarily related *pah* gene clusters found within diverse Proteobacteria, including the *nah* type within *Pseudomonas* ^9^, *nag* type within Burkholderiales^25^, as well as the nitrotoluenes-degradation (*NT*) type within Burkholderiales^26^. Both *nag* and *NT* gene clusters are mainly detected in terrestrial PAHs degraders. In contrast, *nah* genotype is the only PAHs degradation genotype that is widely detected across terrestrial and coastal marine environments^6,7,16,27^. The presence of the *nah* gene clusters in the coastal marine environment makes it possible to study *nah* genes transfer between the terrestrial and marine lineages, as suggested by the *pahAc* fragment discovered in *Marinobacter* sp. NCE312^24^. Unfortunately, there is currently no evidence except the case of *Marinobacter* sp. NCE312 that allow us to address this issue. Therefore, it is worthy to further explore *nah* gene clusters in marine environment and determine whether the horizontal transfer of the *nah* gene clusters or other PAHs degradation genotypes between terrestrial and marine lineages occurs commonly.

In addition, halotolerant enzymes with biotechnological applications from halotolerant or halophilic microorganisms are drawing increasing attentions because of their impressive tolerance to a wide range of salinity^28,29^. Recently, several halotolerant enzymes, including two catechol dioxygenases from *Marinobacter* species, have been characterized^30–32^. However, the halotolerant properties and salt adaption mechanism of catabolic enzymes in PAHs degradation remain unknown although there was one has been cloned from halophilic PAHs degraders^10^.

In this study, we identified *nah*-related PAHs dioxygenases as the dominant genotype in a halophilic phenanthrene-degrading bacterial consortium (CY-1), which is dominated by *Marinobacter* species. *PahAc*s from this consortium form an independent clade in the phylogenetic tree, rather than clustering with any known *nah*-related *pahAc* sequences available in the Genbank. A 5.6 kb PAHs degradation gene cluster belonging to the dominant *nah* group is cloned and the dioxygenase part in this cluster is overexpressed in *E. coli* successfully. Further efforts were made to characterize the property of the PAHs dioxygenase in this halophilic consortium and explore the evolution scenario of the new *pah* gene cluster in this consortium. To date, this is the first report on cloning and overexpression of a halotolerant *nah*-like dioxygenase and prediction of horizontal transfer of *nah*-like gene clusters between halophilic and non-halotolerant bacteria.

## Results and Disscussion

### Microbial community structure in CY-1

**Figure 1:**
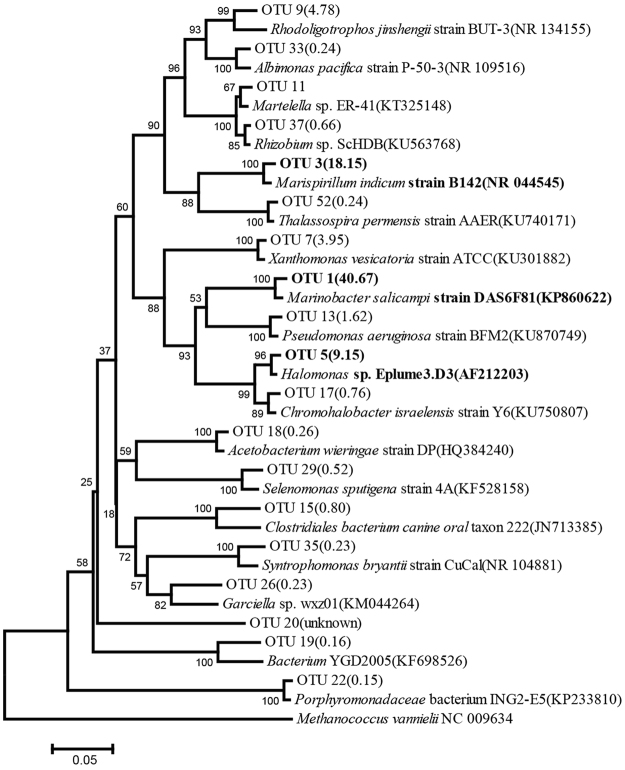
Phylogenetic Neighbor-Joining tree showing the V1 to V3 region of the 16 S rRNA gene sequences of CY-1. The sequences data is separated into different OTUs with 97% identity. The proportions of different genera in CY-1 are shown following the OUT names, and the three most abundant OTUs are shown in bold. For each genus present in the consortium, a reference sequence is included in the phylogenetic tree, and the accession number is shown after the specie names. The percentages of replicate trees in which the associated taxa clustered together in the bootstrap test (1000 replicates) are shown next to the branches. 16 s rRNA gene sequence from *Methanococcus Vannielii* is used as the outgroup in the tree.

The halophilic bacterial consortium CY-1 was enriched by weekly transfers in sea salt-defined media (SSDM) medium^30^ with 10% salinity and phenanthrene (100 mg/L) for 3 months. It could completely degrade 100 mg/L phenanthrene in 6 days. The microbial community structure was determined through high-throughput sequencing (Fig. 1). *Marinobacter* was found to be the most abundant genus and account for 40.67% of the total 16 s rRNA gene sequences, followed by *Marispirillum* (18.15%), and *Halomonas* (9.15%). All of these abundant species were halophiles.

PAHs-degrading strains within genus *Marinobacter* had been isolated from coastal marine environment all around the world^24,33,34^. These *Marinobacter* strains could utilize naphthalene or phenanthrene as the sole carbon source. In a previous study, *Marinobacter* species were also the predominant PAHs degraders in a halophilic phenanthrene-degrading consortium enriched at 10% salinity^35^. The genus *Marispirillum* was identified recently and contained only one *Marispirillum* species, the type strain of which was isolated from an oil-degrading marine consortium but could not degrade PAHs^36^. *Halomonas* species were found in many oil or PAHs-degrading halophilic bacterial consortiums^20,35^; however, none of them were able to degrade PAHs. The consortium also contained several less abundant genera, within which PAHs-degrading strains have been reported previously, i.e. *Pseudomonas* (1.62%), and *Thalassospira* ^37^ (0.24%).

We also tried to isolate pure strains that can grow with phenanthrene as the sole carbon source from CY-1. However, this attempt ended in failure. We did achieve several *Halomonas* strains on the nutrient medium, but we failed to get strains within *Marinobacter* and other genera. In accordance with other previous reports^20,35^, these isolated *Halomonas* strains could not degrade PAHs. Based on the community structure and previous studies^24,35^, the main degraders in this phenanthrene-degrading consortium are most likely to be *Marinobacter* species, while *Halomonas* strains serve as utilizers of intermediates of degradation.

### Profile of PAHs dioxygenase in CY-1

**Figure 2:**
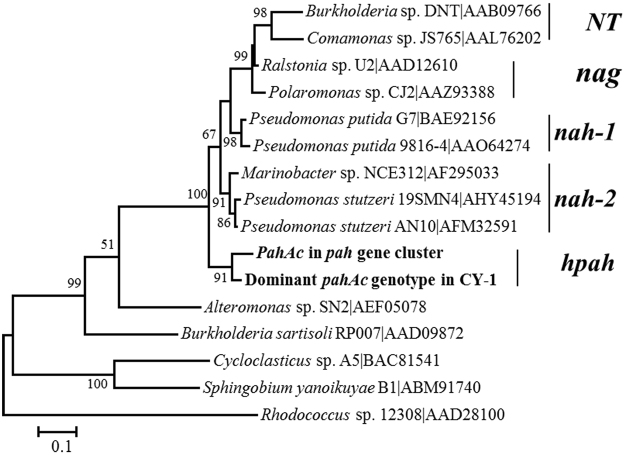
Maximum Likelihood tree of the representative *pahAc* of the dominant genotype in CY-1, the full length *pahAc* gene in the newly-cloned *hpah* gene cluster and other representative *nah*-related *Ac* genes, built with predicted amino acid sequences. Several characterized *pahAc*s from *Alteromonas* sp. SN2, *Burkerholderia* sp. RP007, *Sphingomonas sp*. B1 and *Rhodococcus* sp. NCIB12308 were used as outgroup. *PahAc* sequences cloned in this study are shown in bold. The five types of the *nah*-related *pahAc*s are labeled in the tree, with the group’s name shown in the right of the tree.

Using primers PAH-RHD-396_F_ and PAH-RHD-696R as described previously^7^, the profile of the PAHs dioxygenase in this consortium was investigated. A total of 85 *pahAc* gene sequences were recovered, and corresponded to 7 unique sequences. All of them fell into 3 OTUs when 97% of the amino acid sequence was used as the dividing standard, or one OTU when 90% identity was used. A blast analysis of the representative *pahAc* sequences from CY-1 showed that they were mostly similar to the *nah*-related dioxygenase from *Pseudomonas* and Burkerholderiales. We further built a phylogenetic tree with the representative sequence in CY-1 and other well characterized *nah*-related dioxygenase (Fig. 2). *PahAc* gene sequences cloned from CY-1 formed a single clade, namely *hpcah*, in parallel with the *nah* and *nag* clades in the phylogenetic tree. We also analyzed the phylogeny of all available *nah*-related dioxygenase gene sequences from cultured strains in the Genbank. All previously-reported *nah*-related *pahAc* fell into the three clades of the well-characterized dioxygenases (Fig. S1). These results indicated the dominant dioxygenases in CY-1 represented a new type of *nah*-related PAHs dioxygenases.

### Cloning and sequence analysis of a PAHs-degrading gene cluster

Since different types of *nah*-related clusters vary in structures and PAHs degradation pathways^25,38,39^, it’s attractive to determine the structures of the gene clusters associated with the newly-identified *pahAc*s. After a subsequent gene walking strategy, a 5.6-kb PAH-degradation gene cluster (*hpah*, accession number: KM025345) containing *pahAc* of the dominant type in CY-1 was cloned. A RT-PCR experiment with the total RNA extracted from the phenanthrene-induced CY-1 consortium revealed that this gene cluster was actively transcribed during phenanthrene degradation as shown in Fig. S2.

**Figure 3:**
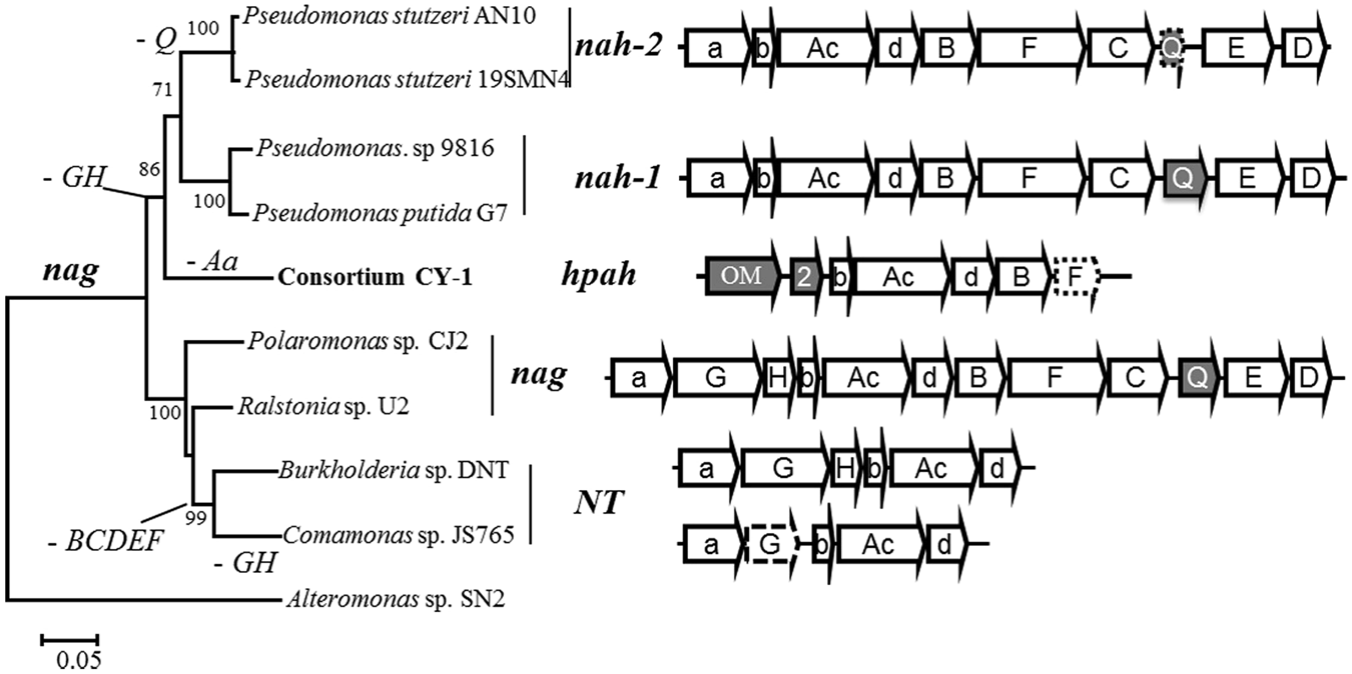
Concatenated tree of *nah*-related *AbAcAdB* genes, organization of *nah*-related gene clusters and proposed evolutionary scenario. On the left is the concatenated tree of *nah*-related *AbAcAdB* genes deduced by both the Maximum Likelihood and Neighbor-Joining method; *AcAdB* genes from *Alteromonas* sp. SN2 were used as the outgroup. On the right are the archetypal structures of the five types of the *nah*-related gene clusters. Arrows in white represent PAHs degradation genes and their transcription directions. For clarity, *nahAaAbAcAd* are shown as *a*, *b*, *Ac*, *d*, etc. Arrows in gray represent genes encoding hypothetical proteins. Arrows with dashed borders represent interrupted genes. The proposed evolutionary events are shown next to the corresponding branches in the tree.

The whole gene cluster contains seven open reading frames (ORFs) (Fig. 3). All except *orf2* are initiated by a canonical ATG start codon and are preceded by a putative ribosomal binding site. Through blast analysis, the five downstream ORFs were found to be most similar to the *nah-related* PAHs degradation genes, namely the *nahAb*, *nahAc*, *nahAd*, *nahB*, and *nahF*, with the highest identities ranging from 81–87% (Table 1). These five ORFs, designed as *hpahAbAcAdBF*, are arranged in the same way as the well-characterized *nah* and *nag* gene clusters^25,38,39^. The homologies of these ORFs and their arrangement in this newly-cloned *hpah* gene cluster suggest that it was evolutionarily-related to the *nah*-related gene clusters.

**Table 1:** Annotation of genes in *hpah* gene cluster.

**Genes**	**Proposed function**	**Protein size**	**Protein with highest identity**	**Identity** (aa)*	Accession no.^a^	Organism
*OM1*	outer membrane protein	461	aromatic hydrocarbon degradation protein	76%	AHI32768	*Marinobacter salaries* R9SW1T
			tbuX, toluene transporter	54%	AAF03168	*Ralstonia pickettii* PK01
			styE, styrene transporter	45%	AAR24508	*Pseudomonas putida* CA-3
			todX, toluene transporter	38%	AAC43318	*Pseudomonas putida* F1
			fadL, Long-chain fatty acid transport protein	23%	P10384	*E. coli* K12
*orf2*		105				
*hpahAb*	ferredoxin	105	naphthalene dioxygenase ferredoxin	87%	AAD02135	*Pseudomonas stutzeri* AN10
*hpahAc*	**RHDs** α subunit	449	Naphthalene 1,2-dioxygenase subunit α	83%	Q51494	*Pseudomonas aeruginosa* PaK1
*hpahAd*	**RHDs** β subunit	194	naphthalene 1,2-dioxygenase	85%	EZQ14081	*Pseudomonas bauzanensis* W13Z2
*hpahB*	cis-naphthalene dihydrodiol dehydrogenase	259	2,3-dihydroxy-2,3-dihydrophenylpropionate	81%	EXF45161	*Pseudomonas sp. BAY1663*
*hpahF*	salicylaldehyde dehydrogenase	452	salicylaldehyde dehydrogenase	86%	4JZ6_A	*Pseudomonas putida* G7

^a^Only sequences with the highest identity to *orf1*, *hpahAb*, *hpahAc*, *hpahAd*, *hpahB*, and *hpahF* from the blast results are shown in this table. *OM1* shares the highest identity with a protein from *Marinobacter salaries* R9SW1T, and several other proteins with known functions are also listed.

The two upstream ORFs, however, exhibit no identities to the upstream genes of the *nah*-related gene clusters. For *orf2*, nothing was returned for the blast analysis with the default setting in GenBank, indicating its low similarity with the existing proteins. The *orf1* (designed as *OM1*) was identified as a FadL-type outer membrane protein (OM), and exhibited the highest identity (75%) with an uncharacterized OM from *Marinobacter salaries* R9SW1T (Table 1). A closer examination of the *OM* gene in *Marinobacter salaries* R9SW1T unveils it is associated with a toluene degradation gene cluster in the genome. Several FadL-type OMs, i.e. TodX^40,41^, TbuX^40,42^, and styE^43^, have been reported to be involved in the degradation of toluene and other aromatics, and were proposed to be responsible for aromatics uptake from the environment. The *OM1* ORF exhibited moderate identity with these proposed aromatics transporters (Table 1), thus was likely involved in PAHs uptake.

In addition, passive diffusion and active transport were two known mechanisms for PAHs uptake by bacteria^44–47^. Because PAHs are poorly soluble in the water, the association of a transport protein to facilitate active PAHs uptake could largely promote PAHs utilization efficiency for PAHs degraders^46^. However, in *Pseudomonas* strains of the *nah* genotype, no potential transporter genes are found in the surrounding regions of the *nah* gene clusters. Accordingly, they are reported to take in PAHs through passive diffusion^44,47^. Currently, the only reported PAHs transporter of all PAHs degraders was an ompW-type OM in a *Pseudomonas* strain, of which the PAHs degradation genotype was undetermined^46^. Thus, we cannot ascertain whether the FadL-type OM associated with this newly-cloned *hpah* gene cluster was actually a PAHs transporter. Anyway, this is the first time to observe a FadL-type OM associated with a *nah-related* gene cluster, which may evolved after horizontal transfer.

### RHD cloning and substrate specificity

Using primer RHD-F and RHD-R, *nahAb/Ac/Ad* genes in this cluster was cloned into the expression vector pET28a (+) and was successfully overexpressed in *E. coli BL21* (DE3). An analysis of crude extracts of IPTG-induced recombinant cells by SDS-PAGE revealed the over-production of two soluble recombinant polypeptides, shown as the first and third stripes (from the top) representing the product of *nahAd* and *nahAc*, respectively (Fig. S2). The *nahAb* product was not detected probably because the standard SDS-PAGE used here was improper for resolving polypeptides in the 10,000- to 15,000-Da range. These results indicated that the RHD were correctly assembled in *E. coli*, which made it possible to analyze its enzymatic characteristics.

We first determined the substrate specificity of this cloned RHD, using GC-MS. As expected, it could readily oxidize naphthalene and phenanthrene into their cis-dihydrodiol forms, with the highest activity towards phenanthrene (Table 2). This RHD could also slightly transform phenol to hydroquinone and oxidize biphenyl Biphenyl cis-2,3-dihydrodiol or n-hydroxy-biphenyl, indicating its monohydroxylation activity. No products were detected for dibenzothiophene and PAHs with four rings, i.e. fluoranthene, pyrene and benz[a]anthracene. Generally, the substrate specificity and the reaction type of this RHD were consistent with other *nah-related* RHDs, which are well known for their ability to oxidize low-molecular-weight PAHs, especially naphthalene and phenanthrene^25,48,49^.

**Table 2:** Substrate specificity of RHD expressed in *E. coli*.

Substrate	Degradation rate(%)	Products	RT(min)	µM Diol/h mgProt^a^
Phenol	8.31	hydroquinone	8.34^c^	0.0045
Biphenyl	12.51	Biphenyl cis-2,3-Dihydrodiol	12.13^c^	0.0043
		4-hydroxy-Biphenyl	9.59^c^	
		2-hydroxy-Biphenyl	11.32^c^	
Dibenzothiophene	6.50	nd	4.20^b^	0.0014
			4.70^b^	
Naphthalene	27.44	Naphthalene 1,2-dihydrodiol	9.75^c^	0.0113
phenanthren	49.93	Phenanthrene cis-3,4-dihydrodiol	14.46^c^	0.0148
Fluoranthene	27.17	nd	5.43^b^	0.0071
Pyrene	1.85	nd	7.92^b^	0.0005
benz[a]anthracene	5.38	nd	5.56^b^	0.0014

^a^Calculated from the HPLC peak areas of biphenyl degraded after 13 h of incubation. The values are averages of three separate determinations with the control data deduced. ^b^HPLC retention time with those of the authentic sample. ^c^GC-MS retention time nd: dibenzothiophene, fluoranthene, pyrene,benz[a]anthracene,benz[a]pyrene, did not give any detectable products.

### Halotolerant properties of the newly-cloned RHD

**Figure 4:**
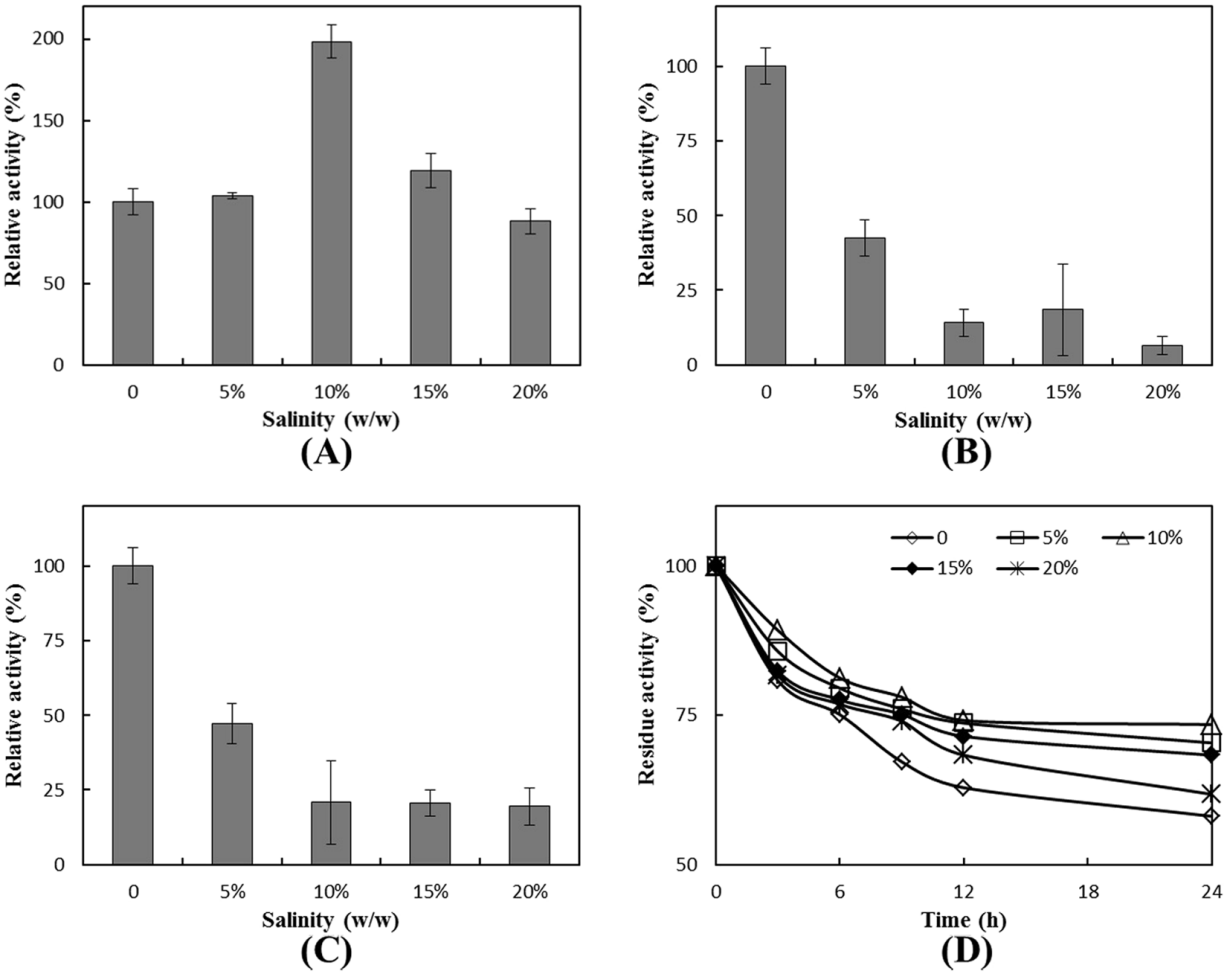
Effects of NaCl concentration on the activity (**A**) and salinity stability (**D**) of RHD obtained from CY-1 and the effect of NaCl concentration on the RHD activity from a non-halotolerant species, *Pseudomonas* (**B**) and *Delftia* sp. Cs1–4 (**C**). Effects of NaCl concentration (**A**, **B** and **C**) was determined at 4 °C in 50 mM PBS buffer (pH = 7.5). Activity was detected by the standard method. The value obtained without NaCl in the reaction mixture was taken as 100%.The enzyme was incubated in sodium phosphate buffers (pH 7.5) and PBS contain 5%, 10%, 15%, 20% NaCl respectively to detect the RHD salinity stability (**D**) and residual activity was determined at 3, 6, 9, 12, 24 h. The values shown represent averages from triplicate experiments. Error bars were not shown in this fig for better looking effect.

This RHD was active over a broad range of NaCl concentrations, as shown in Fig. 4A. It exhibited the highest activity in the PBS medium with 10% NaCl, and could still maintain nearly 100% activity with 20% NaCl, indicating a strong tolerance to NaCl. Furthermore, it could maintain 75% activity after 24 h with 10% NaCl, compared with the 60% activity with 0% NaCl, indicating a higher stability under saline conditions (Fig. 4D).The halotolerant property of this RHD was prominent, especially when compared with another *nah* type dioxygenase (84% identity) from a *Pseudomonas* strain (Fig. 4B) and the PAHs dioxygenase from *Delftia* sp. Cs1–4 (Fig. 4C).

The halotolerant properties of this RHD are similar to that reported for two catechol 2,3-dioxygenases from *Marinobacter* species, all exhibiting higher activity and stability at high NaCl concentration^30^. However, the activity and stability of this RHD were the highest with 10% NaCl and dropped when the concentration of NaCl increased or decreased, while the activity and stability of the two catechol 2,3-dioxygenases remained almost unchanged when the concentration of NaCl fluctuated. In previous studies, several halotolerant enzymes cloned from halotolerant and halophilic bacteria were also reported to exhibit higher activity in saline environment than their non-halotolerant counterparts^31,32^. All of them can maintain activity across a wide range of salinity, from 0% up to 15% or 20%. However, the relationship between the enzymatic activity and salinity varied for different halotolerant enzymes including this RHD.

**Figure 5:**
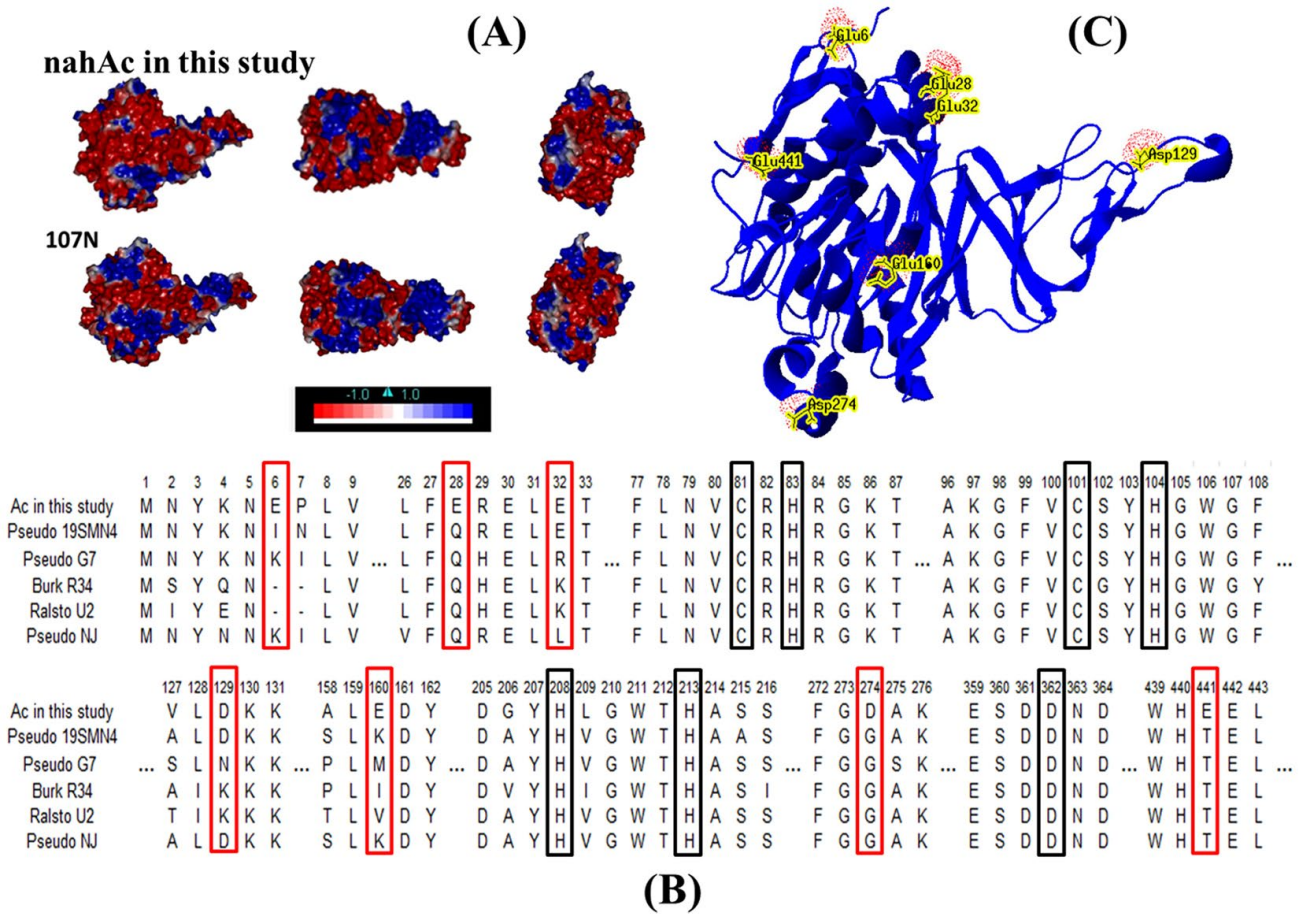
Surface electrostatic potentials analysis. (**A**) Surface electrostatic potentials of nahA3, and Naphthalene 1,2-dioxygenase from *Pseudomonas putida* NCIB 9816–4 (PDB no. 1O7N) as obtained using Discovery Studio 2.5 software. The first, second and third line were seen from the front, top and left of the structures, respectively, with the red surface corresponding to negatively charged residues and the blue surface corresponding to positively charged residues (color figure online). (**B**) The key amino acid sites in nahAc. Fe^2+^ binding sites are labelled in black squares and the sequence near these active sites are also shown. Mutated amino acid sites are labelled in red frame. (**C**) The position of these mutated sites appear at the surface of the large subunit. This graph is illustrated by Swiss-pdb viewer.

Amino acid composition, especially those on the protein surface of the newly-cloned RHD was calculated. Compared with other classic ***nah***-related dioxygenase, this RHD had the highest content of acidic amino acids and the lowest content of alkaline amino acids in the non-conservative region (Table S1). In the large subunit, acidic animo acids at sites 6 (Glu), 28 (Glu), 32(Glu), 129 (Asp), 154 (Asp), 160 (Asp), 263 (Asp) and 274 (Glu) were exclusively found on the surface of this RHD (Fig. 5B,C). Correspondingly, the negative electrostatic surface potential of this RHD was predicted to be much lower than a mesophilic *nah*-related RHD (1O7N, with 83% identity to this RHD) (Fig. 5A). The excess of acidic amino acids were also found on the surface of the small subunit (Ad) of this RHD compared with other ***nah***-related RHD (Table S1). Other fragments in this cluster also contained the higher proportion of acidic amino acids than their mesophilic alleles (Table S1). It might be the high acidic amino acids content at the protein surface that made the *pahAc* obtained from CY-1 present such a high halotolerant property^29,50^.

### Phylogenies of the *hpah* gene cluster

To further elucidate the evolutionary relationships between this *hpah* gene cluster and other *nah*-related gene clusters, we conducted phylogenetic analysis with the *hpah* genes and their alleles in the *nah*-related gene clusters.

In the phylogenetic trees of all genes, *hpah* gene formed an independent branch, in parallel with two *nah* clades (*nah-1* and *nah-2*) and one *nag* clade (including the *NT* clade) (Figs 2, S4–S6). However, the topology of trees differed in the differentiation order of each clade for different genes. For *Ac*, and *B* genes, *hpah* clade first separated from other clades; in contrast, for *Ab*, *Ad* genes, the *nag* clade first separated from other clades. *HpahAb* gene from CY-1 diverged before the divergence of *B* genes in the *nah-1* and *nah-2* clades; in contrast, *hpahAd* gene from CY-1 clustered with the *nah*-2 clade.

The inconsistence of phylogenetic trees of different genes created an obstacle to infer the evolution process of *hpah* gene cluster and other *nah*-related gene clusters. Since genes in these gene clusters were likely to evolve and be transferred as a whole rather than separately, we further constructed a concatenated tree of *AbAcAdB* genes to track the evolution of the whole gene cluster. Interestingly, the topology of the concatenated tree was consistent with the tree of *Ab* genes, indicating *nag* gene clusters first separated from other *nah*-related gene clusters (Fig. 3), followed by the divergence of the newly-identified *hpah* clade and the two *nah* clades from *Pseudomonas*. The concatenated tree was consistent with the organization of the *nah*-related gene clusters (Figs 3, S7), indicating this *hpah* gene cluster represents a new type of *nah*-related gene cluster.

### Evolutionary scenario of *nah*-related gene clusters

The *nah* gene clusters (*nah*_1 in Fig. 3) in *Pseudomonas* were the first PAHs-degradation gene clusters described and are still the best characterized^38,49^. Rafael Bosch *et al*.^39^ revealed the existence of a *nah-related* gene cluster (*nah*_2 type in Fig. 3), in which *nahQ* gene was absent, in *Pseudomonas stutzeri* AN10. In this study, we found the absence of *nahQ* gene was the common feature in all *nah*_2 type gene clusters (Fig. S7), and further analysis revealed a *nahQ* remnant between *nahC* and *nahE* in these gene clusters, suggesting the deletion of *nahQ* was probably the point at which the *nah_*2 gene cluster diverged from the *nah*_1 gene cluster. The *nag* gene cluster was first discovered in *Ralstonia* sp. U2^25^, and was later found in *Polaromonas* ^51^ and *Burkholderia* ^52^. A comprehensive analysis in this study revealed that the *nag* gene clusters were widely distributed in PAH-degrading Burkholderiales (Figs S1, S7). The main difference between the *nag* and *nah* gene clusters was the existence of two genes (*nagG* and *nagH*) between the *nagAa* and *nagAb* genes. *NT* gene clusters in the nitrotoluenes degraders, which were also found mainly in Burkholderiales species, are another type of *nah*-related gene clusters (Figs S1, S6). It is generally accepted that the *NT* gene clusters were derived from the *nag* gene clusters through the elimination of the downstream *nagBFCQED* genes and further deletion of the *nagGH* genes^26^.

The newly-cloned *hpah* gene cluster was clustered with type *nah*−1 and *nah*−2 gene clusters in the phylogenetic tree and did not contain the *G* and *H* genes. It differed from the *nag* and *nah* gene clusters in the absence of *pahAa* gene and the presence of a potential PAHs transporter gene (*OM1*) in the upstream. Because *Aa* gene is present in both *nag* and *nah* gene clusters, *Aa* gene was probably present in the progenitor of *hpah* and *nah* gene clusters and was deleted in the subsequent evolution process of the *hpah* gene cluster. Because *nah* gene clusters have been identified within diverse *Pseudomonas* species and across diverse environments, it is likely that the *hpah* gene cluster was derived from *nah* gene clusters.

The first divergence of the *nah*-related gene clusters occurred between *nag* gene clusters and the progenitor of *nah* and *hpah* gene clusters (Fig. 3). Which is the initial progenitor was yet unsolved. The most obvious difference between these two groups is the *nagGH* genes. In the *nag* gene clusters, products of *nagAa* and *nagAb* genes served as the electron transport components (ETCs) both in salicylate-5-hydroxylating oxygenase coded by *nagGH* genes and in PAHs dioxygenase coded by *nagAcAd* genes^53^. ETC components have been thought to be one of the important selective forces in the evolution of oxygenase^5^. To determine whether *nagAaAb* are the inherent ETCs in salicylate-5-hydroxylating oxygenase or in PAHs dioxygenase may provide insight in the evolution of process the *nag* gene cluster.

**Figure 6:**
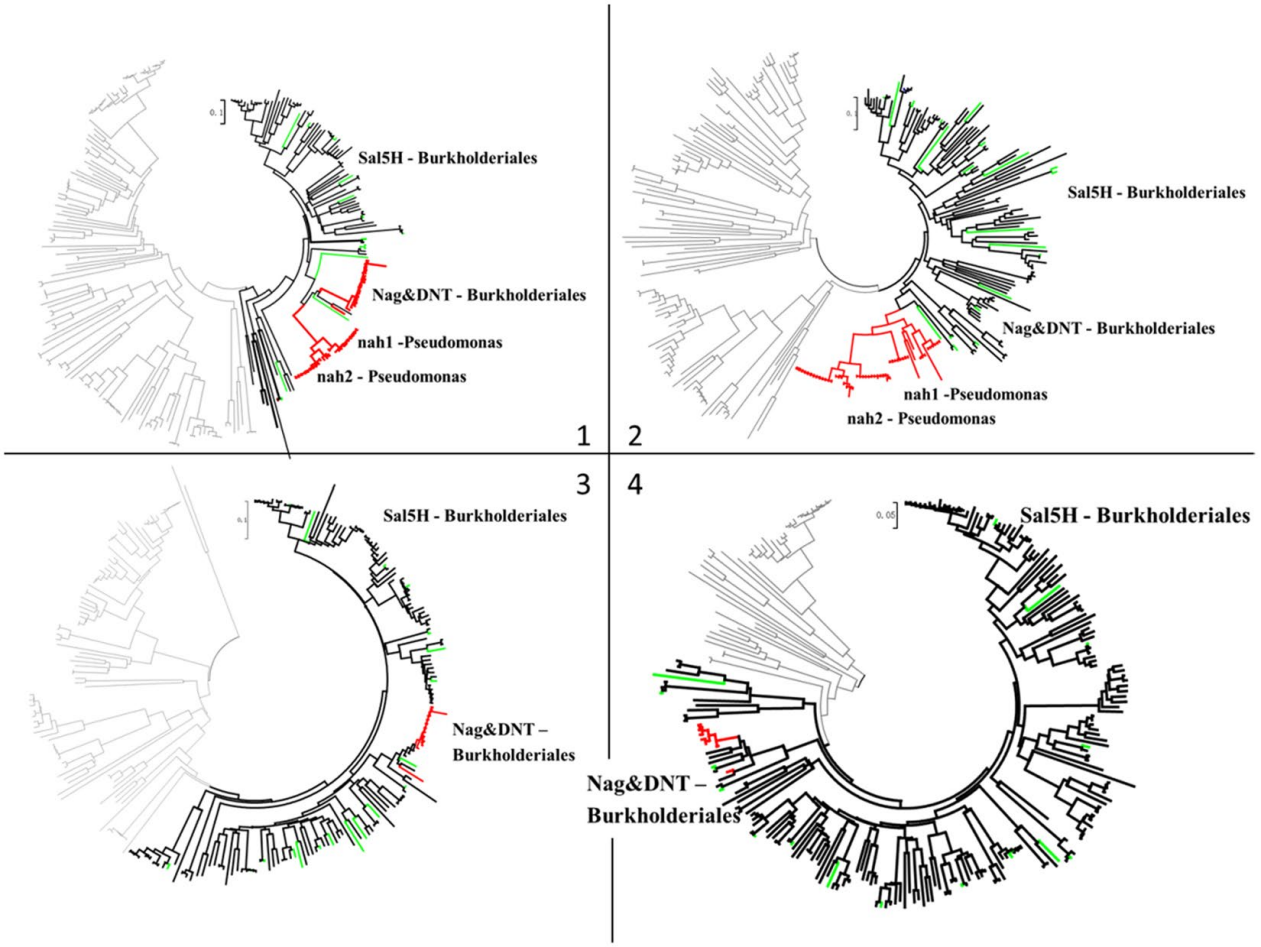
Neighbour-Joining trees for *nagA1* (1), *nagA2* (2), *nagG* (3), *nagH* (4) and their homologous genes. Branches in red are from the *nah*-related gene clusters; branches in bold black are from the Sal5H gene clusters; branches in green are from some representative Sal5H gene clusters whose structures are present in Fig. S7.

Comparison of the phylogenetic trees, built with *Aa*, *Ab*, *Ac*, *Ad*, *G*, and *H* genes and their homologous genes, revealed a similar topology for the trees of *Aa*, *Ab*, *G*, and *H* (Fig. 6). In each tree, genes from *nag* gene clusters formed a single clade surrounded by their homologous alleles mostly from Burkholderiales (Fig. 6). Most of the homologous alleles of *Aa*, *Ab*, *G*, and *H* genes were found to coexist in same strains (Fig. S8). Moreover, these homologous genes were adjacent to each other in the genome (of nearly twenty randomly-chosen strains), i.e., they belonged to the same operon (Fig. S8). These results indicated that *Aa*, *Ab*, *G*, and *H* genes have clustered and coevolved in the salicylate-degradation gene clusters (*sal*) long before they were integrated in the PAHs-degradation gene clusters, i.e. *nagAaAb* are the inherent ETCs in salicylate-5-hydroxylating oxygenase.

Based on the above analysis, the progenitor of *nah*-related gene clusters can be proposed to be a *nag* type gene cluster. Furthermore, it can be concluded that the *nag* gene cluster initially arose in a PAHs-degrading strain within Burholderiales, because the *sal* gene cluster was widely distributed in Burkholderiales, but was rarely found in other bacteria (Figs 6, S8). The *nag* gene cluster was then rampantly transferred across species within Burkholderiales. The following evolutionary scenario of the *nah*-related gene clusters was proposed, as shown in Fig. 3. The *nag* gene clusters are the progenitor of other *nah*-related gene clusters and arose in PAHs-degrading bacteria within Burkerholderales. *NT* gene clusters were derived from the *nag* gene cluster after the deletion of *nagBCDEF* genes and the gradual deletion of the *GH* genes. The *nag* gene cluster was later transferred into other linkages and became the common ancestor of *hpah* gene cluster found within halophiles and of *nah* gene clusters found within *Pseudomonas* species. The *nagGH* genes were deleted subsequently, probably because the central one-ring aromatic intermediate is not gentisate in the new host. *Hpah* gene cluster then separated from *nah* gene clusters, and the *Aa* gene was probably deleted. In *Pseudomonas* strains, the initial *nah* gene cluster further diverged into two groups after the deletion of the useless *Q* gene in the *nah*−2 gene cluster. Although *nah* gene clusters are found mainly in *Pseudomonas*, several gene fragments similar to *nahAc* have been reported in other genera including *Marinobacter* (Fig. S1), suggesting that further inter-genera horizontal gene transfer had taken place. In a word, these *nah*-related gene clusters were evolving to become more compact through elimination of the redundant genes.

Back to *hpah* gene cluster in the halophiles, as mentioned above, it is derived from the *nah*-related gene clusters in the mesophiles via horizontal gene transfer. PAHs degraders with *nag* gene clusters are exclusively isolated from terrestrial environment. Although PAHs degraders with the *nah* gene clusters were found in saline environment, the *nah* type PAHs dioxygenase from *Pseudomonas* sp. strain HY-1 did not possess ability to adapt saline environment in our study. The halotolerant property and structural feature of the *hpah* RHD pointed to the evolutionary process towards adaptation of the saline environment after horizontal gene transfer. Many barriers that can affect the fate of the transferred gene in the new host have been identified or proposed^54^. It have been reported that further evolution process are required for proper work in the new host after horizontal gene transfer for some pathways^55,56^. Microorganisms in marine environment generally have different lifestyles in comparison to the terrestrial microorganisms due to the molecular mechanisms required for adaptation to distinct concentration of salt^23^. The distinct salt concentration can not only cause the segregation of the terrestrial and marine bacteria, but can also cause the inadaptation of gene clusters that undergo horizontal transfer between the terrestrial and marine bacteria. For PAHs degradation genes which are undergoing rampant horizontal transfer, the distinct genotypes present in terrestrial and marine environment just reflect such segregation effects by distinct salt concentration. The newly-discovered *hpah* gene cluster in our study provided the first example of an enzyme that evolved towards salt adaptation after a recent horizontal gene transfer event.

Still much work is required for further understanding this *hpah* gene cluster in CY-1. First, we cannot affirm whether the dominant *pahAc*s and *hpah* gene cluster were actually from *Marinobacter* or from other genera. Taking more efforts to isolate the PAHs-degrading pure strains or sequencing the metagenomics of the consortium are two feasible ways to address this question. Second, the PAHs dioxygenase cloned from this *Marinobacter*-predominant halophilic consortium exhibited remarkable halotolerant capability, similar properties were also reported for two *Marinobacter*-related catechol 2,3-dioxygenase. Whether halotolerant properties present in these enzymes are common feature for the majority of proteins in *Marinobacter* and other halophilic bacteria in the marine environment is an appealing question that requires far more efforts. Moreover, we proposed the newly-cloned *hpah* RHD have gone through an evolution process towards adaptation of saline environment after horizontal gene transfer. It will be attractive to explore whether this process is common for other HGT events between the mesophiles and halophiles. In addition, it is also attractive to further explore whether inadaptation to the distinct salt concentrations is a basic barrier for HGT between the mesophiles and the halophiles, which may serve as an explanation for the distinct PAHs-degradation and other functional genotypes between the terrestrial and marine environments.

## Materials and Methods

### Reagents, strains and plasmids

N-butylboronate (NBB) and antibiotics were obtained from Sigma-Aldrich (United States). PAHs were purchased from Alfa Aesar. Restriction endonucleases, Taq DNA polymerase, DNAiso Reagent, RNAiso Plus, DNase I, cDNA Synthesis Kit, SYBR® Premix Dimer Eraser™ and T4 DNA ligase were from TaKaRa (TAKARA, Dalian, China). pET28a (+) (Invitrogen, Shanghai, China) was used as an expression vector. *E. coli* strains TOP 10 and BL21 (DE3) (Qiagene, Beijing, China) were used for general cloning and protein expression.

*Pseudomonas* sp. strain HY-1 is isolated from oil-polluted soil and maintained using a mixture of phenanthrene and naphthalene as the sole carbon source in our lab; its 16 S gene sequence has been deposited in GenBank (accession number: MF689060). *Delftia* sp. strain Cs1–4 was kindly denoted by William J. Hickey’lab, and has been reported previously^12^.

### Bacterial consortium and growth conditions

A halophilic bacteria consortium CY-1 capable of degrading phenanthrene under 10% salinity (w/w) was cultured using sea salt-defined media (SSDM) medium under 30 degrees, 150 rpm as described previously^30^.

### PAH-degrading gene cluster amplification and sequencing analysis

#### DNA extraction, consortium structure and profile of PAHs dioxygenase

DNA extraction, consortium structure and profile of PAHs dioxygenase. The genome DNA from the enrichment was extracted by DNeasy Blood & Tissue Kit (Qiagen, Germany) with the process according to manufacturer’s instructions.

The consortium structure in CY-1 was investigated by 454-pyrosequencing following Polymerase Chain Reaction (PCR) strategy as previously described. The RHD diversity was investigated using primers PAH-RHD-396_F_ (5′-ATTGCGCTTAYCAYGGBTGG-3′) and PAH-RHD-696R (5′-ATAGGTGTCTCCAACRAARTT-3′) as described previously^7^. A clone library of *pahAc*s was then constructed in *E. coli*. A total of one hundred clones were selected for sequencing, and 85 *pahAc* gene sequences were recovered.

#### Gene cluster amplification and annotation

The first fragment containing the naphthalene dioxygenase was achieved by PCR using the degenerate primers as described by Gomes^57^. The other fragments contained in the whole PAHs degradation cluster were obtained by thermal asymmetric interlaced PCR (Tail-PCR) according to the manufacturer’s instructions, using different sets of divergent primers designed on the basis of step by step sequences. The amplified gene cluster were completely sequenced and uploaded in GenBank (Accession no KM025345). Gene annotation and similarity analysis were carried out using the NCBI Web BLAST service^58^.

#### Phylogenetic analysis

To collect all the *nah-related pahAc* gene sequences and their corresponding gene clusters that are available in the GenBank database, amino acid sequence of the *hpahAc* cloned in this study is used to blast against the Non-redundant protein database using the NCBI Web BLAST service^58^, and 5000 sequences with the lowest e-values from cultured microorganisms are retrieved. With the retrieved sequences, a preliminary distance tree was created by using the NCBI tree viewer tool with the default parameters on the NCBI Web BLAST service^58^. All *pahAc* sequences that fell into the *nah*-related clade of the distance tree were collected for further analysis. The corresponding gene clusters of the *nah*-related *pahAc*s, if existing, are collected. Phylogenetic tree of each gene was then created by using the Maximum Likelihood method and the neighbor-joining method. Total bootstrap value was 100 in each tree. To construct the concatenated tree of *hpahAbAcAdB* genes, sequences of each gene were first aligned; and the aligned gene sequences from the same bacterium were then concatenated; the concatenated sequence for each bacterium was then used to construct the final phylogenetic tree in the same way as the individual genes. All phylogenetic analyses were conducted in MEGA6^59^.

### RHD cloning, overexpression and characterization

#### Cloning and overexpression

The *nahAbAcAd* fragment encoding RHD in the whole cluster was amplified by PCR using primers pairs: RHD-F (5′-GCGCATATGATGACAGAGAAGTGGATC-3′) and RHD-R (5′-CGGAGCTCTTACAGAAAGACCATTACG-3′), introducing the *Nde I* and *Sac I* (in underlined) at the end of primer. PCR products were ligated into pMD-19T, sequenced and then sub-cloned into the *Nde I* and *Sac I* sites of expression vector pET28a (+) (Novagen, USA). The construct was transformed into *E. coli* strain BL21 (DE3) for expression analysis.

#### Sodium dodecyl sulfate-polyacrylamide gel electrophoresis (SDS-PAGE)

Bacterial cells were pelleted by centrifugation and washed with phosphate buffer (pH 7.5). One milliliter of phosphate buffer was added to the pellet and the suspension was then subjected to sonication on ice for 2 min (5 s pulse interval; 40% of maximum amplitude). After centrifugation, the supernatant and the pellet were detected by SDS-PAGE. After electrophoresis, the gel was stained with Coomassie Brilliant Blue. The molecular mass of the protein was determined by comparison of the control.

#### RT-PCR analysis of RHD gene

Real-time PCR analysis was performed using the SYBR Green-based detection system to monitor RHD gene expression. Cells were harvested at 0, 4, 8, 12, 16, 24, 36, 48, 72 and 96 h by centrifugation at 10,000 g at 4 °C for 1 min. Total DNA and RNA were extracted directly from each culture using the DNAiso Reagent and RNAiso Plus. The RNA was treated with RNase-free DNase according to the manufacturer’s instructions. Reverse transcription of RNA samples was performed using the Prime Script 1st strand cDNA synthesis Kit.

RHD genes and transcripts were quantified in an iCycleriQ (Bio-Rad, America) with the primer pairs as follow: *nahAd*-5′-GGTTGTGTCACGGGAACTG-3′, *nahAd*-5′-GATCAGGTTGGAGCGAACG-3′. At the end of the Real-Time PCR a melting curve analysis was performed by a final step that consisted of measurement of the SYBR Green I signal intensities during a 0.5 °C temperature increment every 10 s from 51 °C to 95 °C. The standard curve for gene quantification was performed with the *nahAd* PCR product cloned in the pMD-19T TA cloning kit. The gene expression level was calculated based on the transcript/gene ratio.

#### Substrates preference of the RHD

The activity of recombinant *nahAb/Ac/Ad* expressed in *E. coli* was assayed as follows. Strains BL21 (DE3) (pET28a) were grown in Luria-Bertani medium containing suitable antibiotics to an OD600 of 0.6 and then incubated at 30 °C with 0.5 mM IPTG. After 5 hours, cells were centrifuged, washed and resuspended to an OD600 of 1.2 in M9 minimal medium containing 0.2% glucose, and distributed as 5 ml aliquots into Erlenmeyer flasks. Fifty microliters of PAHs, including phenol, biphenyls, naphthalene, phenanthrene, dibenzothiophene, fluoranthene, pyrene, benz[a]anthracene and benzo[a]pyrene, dissolved in N,N-dimethyl formamide (stock 100 mg/ml) were added to the sterile liquid medium. The flasks were incubated at 150 rpm for 13 h at 30 °C. All the tests were performed in triplicates.

Water-soluble products resulting from PAHs oxidation were extracted with ethyl acetate, dried over anhydrous Na_2_SO_4_ and evaporated under nitrogen gas. The dried samples were dissolved in 500 μl acetonitrile for GC-MS (Agilent 7890 A GC, Inert MSD with TripleAxis Detector, Agilent, USA) analyses.

PAHs and their degradation products were analyzed by reversed phase HPLC with UV detection at 254 nm. The mobile phase, supplied at 1 ml min^−1^, was a 10 min linear gradient from 60% (v/v) to 90% (v/v) aqueous methanol with holding at 90% aqueous methanol for 10 min. The analytical column was a 4.6 × 250-mm, 5-μm C_18_ Inertsil ODS-3 column (Beijing Mairuida Technology, Beijing, China).

Before GC-MS analysis, the extracts were derivatized either with bis (trimethysilyl) trifluoroacetamide: trimethylchlorosilane (99:1) or NBB, according to the manufacturer′s instructions. An HP-5 capillary column (30 m × 0.25 mm I.D. × 0.25 µm film thickness) was used to separate the products. The column temperature was programmed as follows: started at 60 °C and held for 2 min, ramped at 10 °C/min to 280 °C, and then held for 10 min. Helium gas was used as the carrier gas at a flow rate of 1 ml/min. The mass spectrometer was operated in the mass scan mode.

#### Enzyme assays and effects of NaCl on RHD activity

Cells induced by 0.5 mM IPTG were disrupted by sonication, the cellular debris was removed by centrifugation at 14,000 g for 20 min, and the supernatant was used as the crude cell extract. Dioxygenase activity was assayed through the transform of naphthalene by HPLC.

RHD characteristics were measured after the enzyme incubated under different conditions such as a wide range of salinity and pH for 30 min. The RHD temperature stability was measured after incubation under correspond temperatures for 3 h, 6 h, 9 h, 12 h and 24 h. The RHD salinity stability was measured after incubation under correspond salinities for 3 h, 6 h, 9 h, 12 h and 24 h at 4 °C.

#### Surface electrostatic potential

The surface electrostatic potential of the RHD obtained in CY-1 and a mesophilic homolog obtained from protein database (PDB no. 1O7N) was calculated by Discovery Studio 2.5 software (Accelrys, SanDiego, CA, USA).

## Electronic supplementary material

Supplemental Material
