# Supplementary material for: Salt Adaptation and Evolutionary Implication of a Nah-related PAHs Dioxygenase cloned from a Halophilic Phenanthrene Degrading Consortium
Source: Sci Rep. 2017 Oct 2;7:12525. doi: 10.1038/s41598-017-12979-z (PMC5624874; doi:10.1038/s41598-017-12979-z)
Supplement: Supplementary file 1 — Supplemental Material [file 41598_2017_12979_MOESM1_ESM.pdf]

Salt Adaptation and Evolutionary Implication of a *Nah*-related PAHs Dioxygenase cloned from a Halophilic Phenanthrene Degrading Consortium

Chongyang Wang, Guang Guo, Yong Huang, Han Hao, Hui Wang *

State Key Joint Laboratory of Environment Simulation and Pollution Control, School of Environment, Tsinghua University, Beijing, 100084, China

* Correspondence to Hui Wang: wanghui@mail.tsinghua.edu.cn.

Tel: 86-10-62772137; Fax: 86-10-62771472;

- Chongyang Wang, 13051971091@163.com;
- Guang Guo, guoguang007007@163.com;

  Present address: School of Environmental Engineering, Nanjing Institute of Technology, Nanjing 211167, China
- Yong Huang, hy791441103@yeah.net;

Table S1 show the amino acid constitution of non-conservative region of *nahAc*, *nahAd*, *nahAb*, and *nahB*

Figure S1 show the phylogenetic tree of all *nah*-related *Ac* sequences from cultured bacteria in GenBank

Figure S2 show the relative expression level of the *nahAd* gene in the *pah* gene cluster after induction with phenanthrene.

Figure S3 show the detection of nahAbAcAd overproduced in *E. coli* BL21 (DE3)

Figure S4-S6 show the phylogenetic trees of *nah*-related *Ab, Ad, B* genes, respectively

Figure S7 show the gene organization in all *nah*-like gene clusters available in GenBank

Figure S8 show the gene organization in the representative gentisate gene clusters

Table. S1 Amino Acid constitution of non-conservative region in *nah*-related *Ab, Ac, Ad*, *B* genes

Strain	*nahAb*		*nahAc*		*nahAd*		*nahB*	
	Acid	Basic	Acid	Basic	Acid	Basic	Acid	Basic
***Pah* gene cluster in this study**	**19.57**	**8.70**	**14.79**	**11.24**	**14.12**	**9.41**	**13.58**	**19.75**
Pseudo G7	17.39	8.70	10.65	12.43	8.14	12.79	12.05	20.48
Coma H	15.22	13.04	10.78	13.17	8.14	11.63	10.84	20.48
uncultured	13.04	8.70	10.65	11.83	8.14	12.79	12.05	20.48
Pseudo C18	13.04	8.70	10.65	11.83	6.98	11.63	12.05	21.69
Pseudo 9816	13.04	8.70	10.65	11.83	6.98	11.63	12.05	21.69
Pseudo PC20	13.04	8.70	10.65	11.83	6.98	11.63	12.05	21.69
Pseudo BS202	13.04	8.70	10.65	11.83	6.98	11.63	12.05	21.69
Ralsto U2	15.22	13.04	10.78	13.17	8.14	12.79	10.84	19.28
Burk C3	15.22	10.87	10.18	13.17	8.14	12.79	9.64	20.48
Pseudo OUS82	19.57	8.70	9.47	11.83	9.30	11.63	12.05	20.48
Pseudo PaK1	15.22	8.70	12.43	13.61	12.94	9.41	9.64	20.48
Polaro CJ2	15.22	8.70	10.18	11.98	6.98	13.95		
Burk R34	15.22	10.87	10.18	11.38	8.14	13.95		
Diapho DS2	15.22	10.87	9.58	12.57	9.41	11.76		
Diapho DS3	15.22	10.87	9.58	12.57	9.41	11.76		
Coma JS765	15.22	10.87	8.98	12.57	9.30	13.95		
**average**	**15.22**	**9.85**	**10.64**	**12.29**	**8.72**	**12.07**	**11.57**	**20.72**

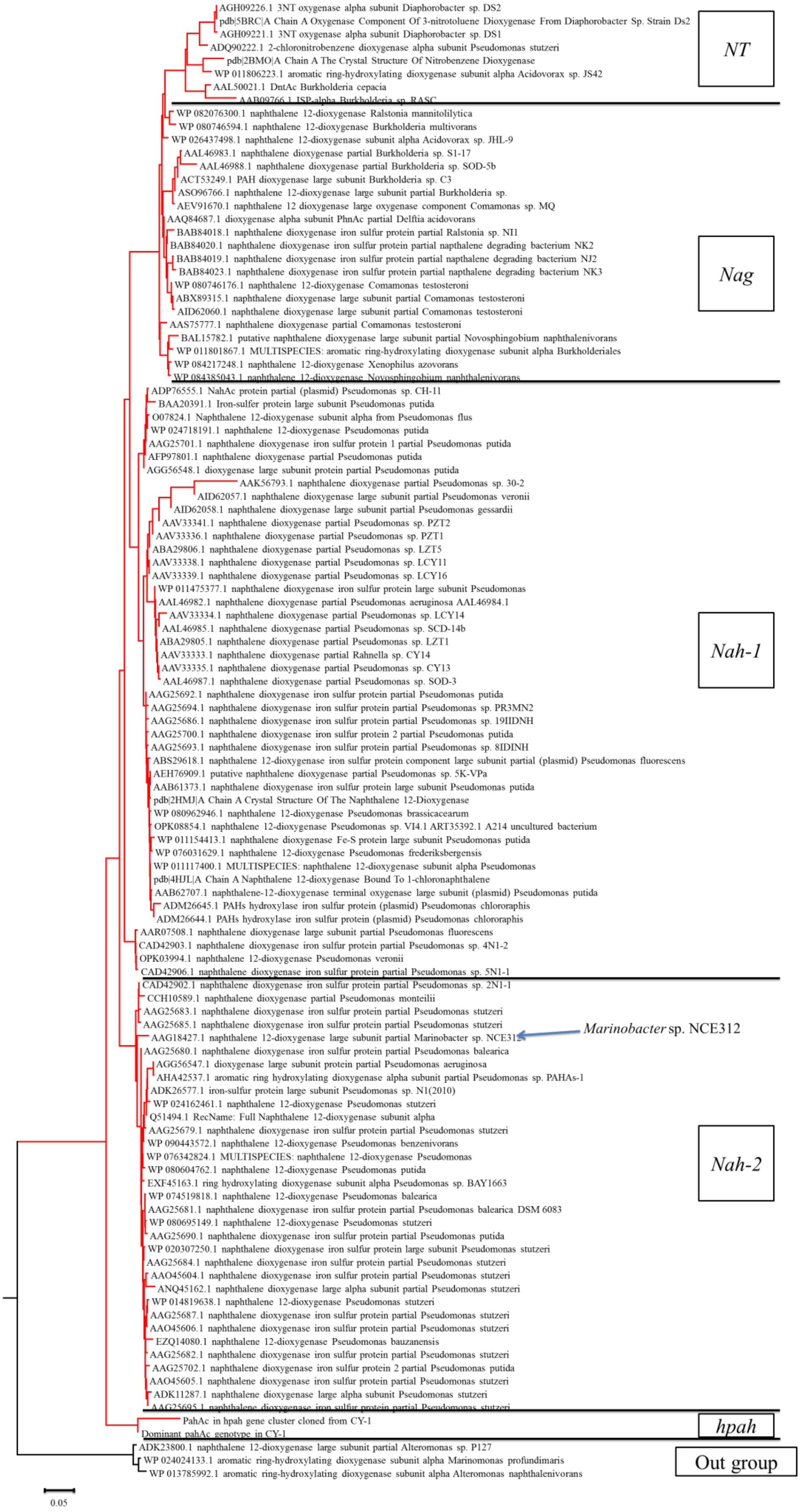
AGH09226.1 3NT oxygenase alpha subunit Diaphorobacter sp. DS2
pdb|5BRC|A Chain A Oxygenase Component Of 3-nitrotoluene Dioxygenase From Diaphorobacter Sp. Strain Ds2
AGH09221.1 3NT oxygenase alpha subunit Diaphorobacter sp. DS1
ADQ90222.1 2-chloronitrobenzene dioxygenase alpha subunit Pseudomonas stutzeri
pdb|2BMO|A Chain A The Crystal Structure Of Nitrobenzene Dioxygenase
WP 011806223.1 aromatic ring-hydroxylating dioxygenase subunit alpha Acidovorax sp. JS42
AAL50021.1 DntAc Burkholderia cepacia
AAB09766.1 ISP-alpha Burkholderia sp. RASC
NT
WP 082076300.1 naphthalene 12-dioxygenase Ralstonia mannitolilytica
WP 080746594.1 naphthalene 12-dioxygenase Burkholderia multivorans
WP 026437498.1 naphthalene 12-dioxygenase subunit alpha Acidovorax sp. JHL-9
AAL46983.1 naphthalene dioxygenase partial Burkholderia sp. S1-17
AAL46988.1 naphthalene dioxygenase partial Burkholderia sp. SOD-5b
ACT53249.1 PAH dioxygenase large subunit Burkholderia sp. C3
ASO96766.1 naphthalene 12-dioxygenase large subunit partial Burkholderia sp.
AEV91670.1 naphthalene 12 dioxygenase large oxygenase component Comamonas sp. MQ
AAQ84687.1 dioxygenase alpha subunit PhnAc partial Delftia acidovorans
BAB84018.1 naphthalene dioxygenase iron sulfur protein partial Ralstonia sp. NI1
BAB84020.1 naphthalene dioxygenase iron sulfur protein partial napthalene degrading bacterium NK2
BAB84019.1 naphthalene dioxygenase iron sulfur protein partial napthalene degrading bacterium NJ2
BAB84023.1 naphthalene dioxygenase iron sulfur protein partial napthalene degrading bacterium NK3
WP 080746176.1 naphthalene 12-dioxygenase Comamonas testosteroni
ABX89315.1 naphthalene dioxygenase large subunit partial Comamonas testosteroni
AID62060.1 naphthalene dioxygenase large subunit partial Comamonas testosteroni
AAS75777.1 naphthalene dioxygenase partial Comamonas testosteroni
BAL15782.1 putative naphthalene dioxygenase large subunit partial Novosphingobium naphthalenivorans
WP 011801867.1 MULTISPECIES: aromatic ring-hydroxylating dioxygenase subunit alpha Burkholderiales
WP 084217248.1 naphthalene 12-dioxygenase Xenophilus azovorans
WP 084385043.1 naphthalene 12-dioxygenase Novosphingobium naphthalenivorans
Nag
ADP76555.1 NahAc protein partial (plasmid) Pseudomonas sp. CH-11
BAA20391.1 Iron-sulfer protein large subunit Pseudomonas putida
O07824.1 Naphthalene 12-dioxygenase subunit alpha from Pseudomonas flus
WP 024718191.1 naphthalene 12-dioxygenase Pseudomonas putida
AAG25701.1 naphthalene dioxygenase iron sulfur protein 1 partial Pseudomonas putida
AFP97801.1 naphthalene dioxygenase partial Pseudomonas putida
AGG56548.1 dioxygenase large subunit protein partial Pseudomonas putida
AAK56793.1 naphthalene dioxygenase partial Pseudomonas sp. 30-2
AID62057.1 naphthalene dioxygenase large subunit partial Pseudomonas veronii
AID62058.1 naphthalene dioxygenase large subunit partial Pseudomonas gessardii
AAV33341.1 naphthalene dioxygenase partial Pseudomonas sp. PZT2
AAV33336.1 naphthalene dioxygenase partial Pseudomonas sp. PZT1
ABA29806.1 naphthalene dioxygenase partial Pseudomonas sp. LZT5
AAV33338.1 naphthalene dioxygenase partial Pseudomonas sp. LCY11
AAV33339.1 naphthalene dioxygenase partial Pseudomonas sp. LCY16
WP 011475377.1 naphthalene dioxygenase iron sulfur protein large subunit Pseudomonas
AAL46982.1 naphthalene dioxygenase partial Pseudomonas aeruginosa AAL46984.1
AAV33334.1 naphthalene dioxygenase partial Pseudomonas sp. LCY14
AAL46985.1 naphthalene dioxygenase partial Pseudomonas sp. SCD-14b
ABA29805.1 naphthalene dioxygenase partial Pseudomonas sp. LZT1
AAV33333.1 naphthalene dioxygenase partial Rahnella sp. CY14
AAV33335.1 naphthalene dioxygenase partial Pseudomonas sp. CY13
AAL46987.1 naphthalene dioxygenase partial Pseudomonas sp. SOD-3
AAG25692.1 naphthalene dioxygenase iron sulfur protein partial Pseudomonas putida
AAG25694.1 naphthalene dioxygenase iron sulfur protein partial Pseudomonas sp. PR3MN2
AAG25686.1 naphthalene dioxygenase iron sulfur protein partial Pseudomonas sp. 19IIDNH
AAG25700.1 naphthalene dioxygenase iron sulfur protein 2 partial Pseudomonas putida
AAG25693.1 naphthalene dioxygenase iron sulfur protein partial Pseudomonas sp. 8IDINH
ABS29618.1 naphthalene 12-dioxygenase iron sulfur protein component large subunit partial (plasmid) Pseudomonas fluorescens
AEH76909.1 putative naphthalene dioxygenase partial Pseudomonas sp. 5K-VPa
AAB61373.1 naphthalene dioxygenase iron sulfur protein large subunit Pseudomonas putida
pdb|2HMJ|A Chain A Crystal Structure Of The Naphthalene 12-Dioxygenase
WP 080962946.1 naphthalene 12-dioxygenase Pseudomonas brassicacearum
OPK08854.1 naphthalene 12-dioxygenase Pseudomonas sp. VI4.1 ART35392.1 A214 uncultured bacterium
WP 011154413.1 naphthalene dioxygenase Fe-S protein large subunit Pseudomonas putida
WP 076031629.1 naphthalene 12-dioxygenase Pseudomonas frederiksbergensis
WP 011117400.1 MULTISPECIES: naphthalene 12-dioxygenase subunit alpha Pseudomonas
pdb|4HJL|A Chain A Naphthalene 12-dioxygenase Bound To 1-chloronaphthalene
AAB62707.1 naphthalene-12-dioxygenase terminal oxygenase large subunit (plasmid) Pseudomonas putida
ADM26645.1 PAHs hydroxylase iron sulfur protein (plasmid) Pseudomonas chlororaphis
ADM26644.1 PAHs hydroxylase iron sulfur protein (plasmid) Pseudomonas chlororaphis
AAR07508.1 naphthalene dioxygenase large subunit partial Pseudomonas fluorescens
CAD42903.1 naphthalene dioxygenase iron sulfur protein partial Pseudomonas sp. 4N1-2
OPK03994.1 naphthalene 12-dioxygenase Pseudomonas veronii
CAD42906.1 naphthalene dioxygenase iron sulfur protein partial Pseudomonas sp. 5N1-1
Nah-1
CAD42902.1 naphthalene dioxygenase iron sulfur protein partial Pseudomonas sp. 2N1-1
CCH10589.1 naphthalene dioxygenase partial Pseudomonas monteilii
AAG25683.1 naphthalene dioxygenase iron sulfur protein partial Pseudomonas stutzeri
AAG25685.1 naphthalene dioxygenase iron sulfur protein partial Pseudomonas stutzeri
AAG18427.1 naphthalene 12-dioxygenase large subunit partial Marinobacter sp. NCE312
Marinobacter sp. NCE312
AAG25680.1 naphthalene dioxygenase iron sulfur protein partial Pseudomonas balearica
AGG56547.1 dioxygenase large subunit protein partial Pseudomonas aeruginosa
AHA42537.1 aromatic ring hydroxylating dioxygenase alpha subunit partial Pseudomonas sp. PAHAs-1
ADK26577.1 iron-sulfur protein large subunit Pseudomonas sp. N1(2010)
WP 024162461.1 naphthalene 12-dioxygenase Pseudomonas stutzeri
Q51494.1 RecName: Full Naphthalene 12-dioxygenase subunit alpha
AAG25679.1 naphthalene dioxygenase iron sulfur protein partial Pseudomonas stutzeri
WP 090443572.1 naphthalene 12-dioxygenase Pseudomonas benzenivorans
WP 076342824.1 MULTISPECIES: naphthalene 12-dioxygenase Pseudomonas
WP 080604762.1 naphthalene 12-dioxygenase Pseudomonas putida
EXF45163.1 ring hydroxylating dioxygenase subunit alpha Pseudomonas sp. BAY1663
WP 074519818.1 naphthalene 12-dioxygenase Pseudomonas balearica
AAG25681.1 naphthalene dioxygenase iron sulfur protein partial Pseudomonas balearica DSM 6083
WP 080695149.1 naphthalene 12-dioxygenase Pseudomonas stutzeri
AAG25690.1 naphthalene dioxygenase iron sulfur protein partial Pseudomonas putida
WP 020307250.1 naphthalene dioxygenase iron sulfur protein large subunit Pseudomonas stutzeri
AAG25684.1 naphthalene dioxygenase iron sulfur protein partial Pseudomonas stutzeri
AAO45604.1 naphthalene dioxygenase iron sulfur protein partial Pseudomonas stutzeri
ANQ45162.1 naphthalene dioxygenase large alpha subunit partial Pseudomonas stutzeri
WP 014819638.1 naphthalene 12-dioxygenase Pseudomonas stutzeri
AAG25687.1 naphthalene dioxygenase iron sulfur protein partial Pseudomonas stutzeri
AAO45606.1 naphthalene dioxygenase iron sulfur protein partial Pseudomonas stutzeri
EZQ14080.1 naphthalene 12-dioxygenase Pseudomonas bauzanensis
AAG25682.1 naphthalene dioxygenase iron sulfur protein partial Pseudomonas stutzeri
AAG25702.1 naphthalene dioxygenase iron sulfur protein 2 partial Pseudomonas putida
AAO45605.1 naphthalene dioxygenase iron sulfur protein partial Pseudomonas stutzeri
ADK11287.1 naphthalene dioxygenase large alpha subunit Pseudomonas stutzeri
AAG25695.1 naphthalene dioxygenase iron sulfur protein partial Pseudomonas stutzeri
Nah-2
PahAc in hpah gene cluster cloned from CY-1
Dominant pahAc genotype in CY-1
hpah
ADK23800.1 naphthalene 12-dioxygenase large subunit partial Alteromonas sp. P127
WP 024024133.1 aromatic ring-hydroxylating dioxygenase subunit alpha Marinomonas profundimaris
WP 013785992.1 aromatic ring-hydroxylating dioxygenase subunit alpha Alteromonas naphthalenivorans
Out group
0.05

Fig. S1 Phylogenetic tree of all *nah*-related *Ac* sequences from cultured bacteria in GenBank. This tree is directly output from the distance tree of the blast result in GenBank. Separation of the four *nah*-related genotypes, i.e. *nah-1, nah-2, nag, NT* and *hpah*, were labeled in the tree.

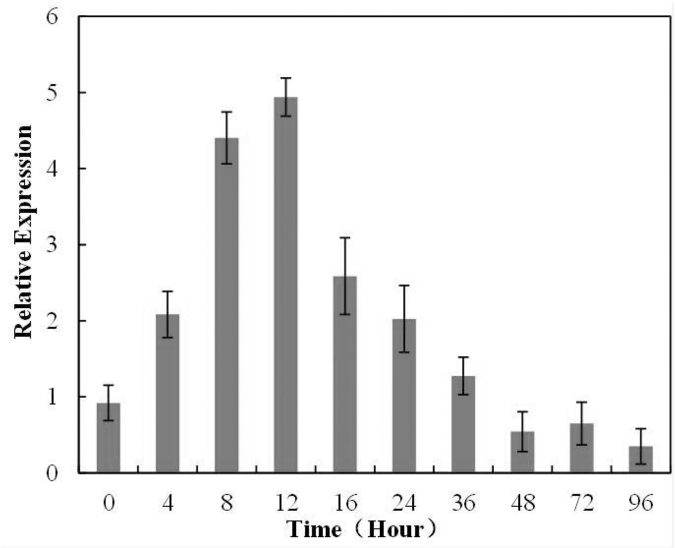

Fig. S2 Relative expression level of the *nahAd* genes after induction.The transcript/gene ratio during microcosm incubation was calculated. Gene expression was quantified using qRT-PCR and the comparative critical threshold ($2^{\Delta\Delta CT}$) method. Three measure replicates and three microcosm replicates were performed. Vertical bars represent standared deviations of the nine replicates of *nahAd* gene quantifications.

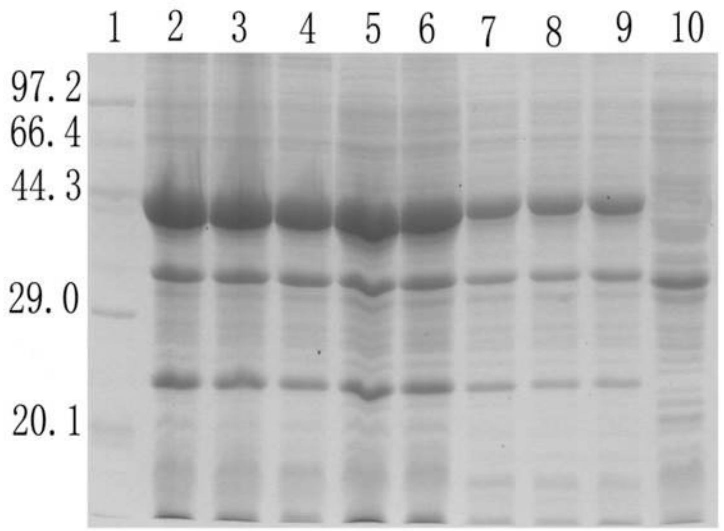

Fig. S3 Detection of nahAbAcAd overproduced in *E. coli* BL21 (DE3) (pET 28a). High amounts of 50 and 20 kDa that were mainly soluble.

1: maker; 2: 30°C 1 mM IPTG; 3: 30°C 0.75 mM IPTG; 4: 30°C 0.5 mM IPTG; 5: 25°C 1 mM; 6: IPTG 25°C 0.5 mM IPTG; 7: 25°C 0.75 mM IPTG; 8: 20°C 0.5 mM IPTG; 9: 20°C 1 mM IPTG; 10: *E. coli* BL21 (DE3) harboring pET28(a)

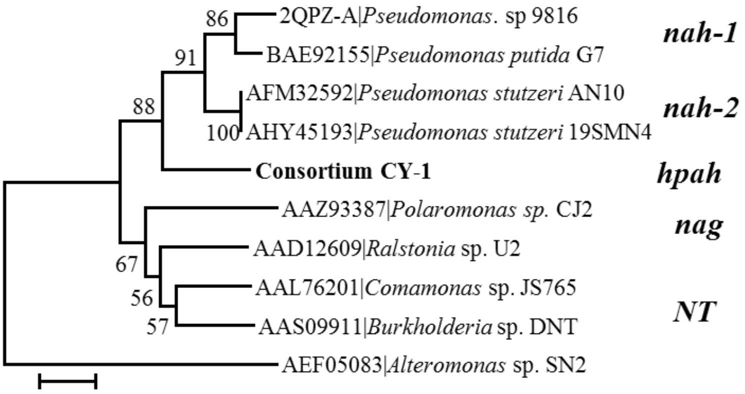

Fig. S4 Neighbor-joining phylogenetic tree established based on the predicted amino acid sequence of *pahAb* and other *nahAb* genes. The phylogenetic and molecular evolutionary analyses are conducted by MEGA 6. All the sequences are obtained from GenBank and the accession number are shown ahead the strain names. Different groups are classified as Fig. 2.

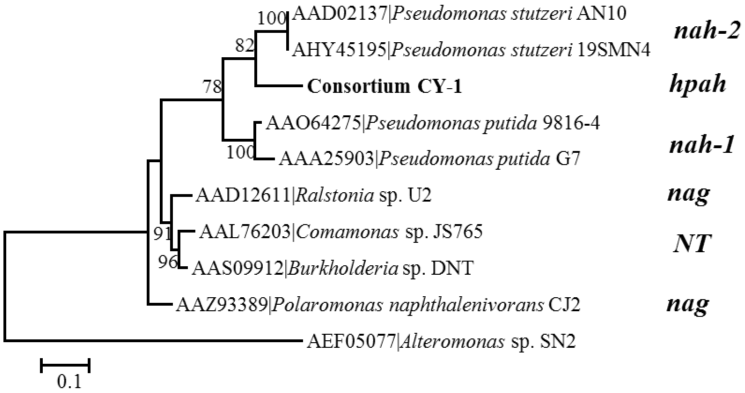

Fig. S5 Neighbor-joining phylogenetic tree established based on the predicted amino acid sequence of *pahAd* and other *nahAd* dioxygenase. The phylogenetic and molecular evolutionary analyses are conducted by MEGA 6. All the sequences are obtained from GenBank and the accession numbers are shown ahead the strain names. Different groups are classified as Fig. 2.

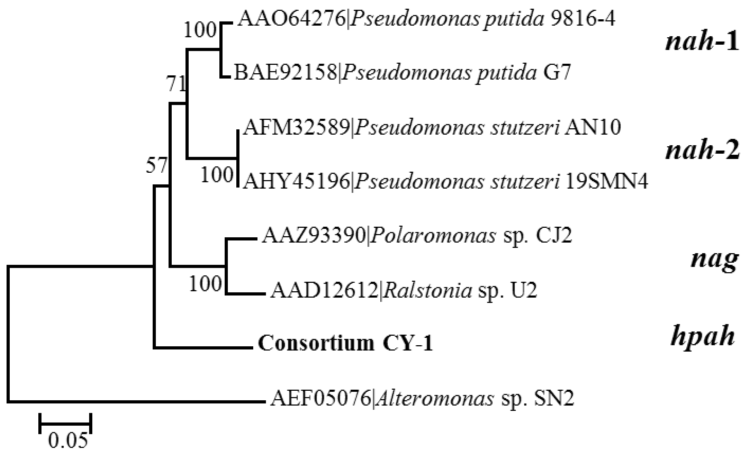

Fig. S6 Neighbor-joining phylogenetic tree established based on the predicted amino acid sequence of *pahB* and other *nahB* genes. The phylogenetic and molecular evolutionary analyses are conducted by MEGA 6. All the sequences are obtained from GenBank and the accession numbers are shown ahead the strain names. Different groups are classified as Fig. 2.

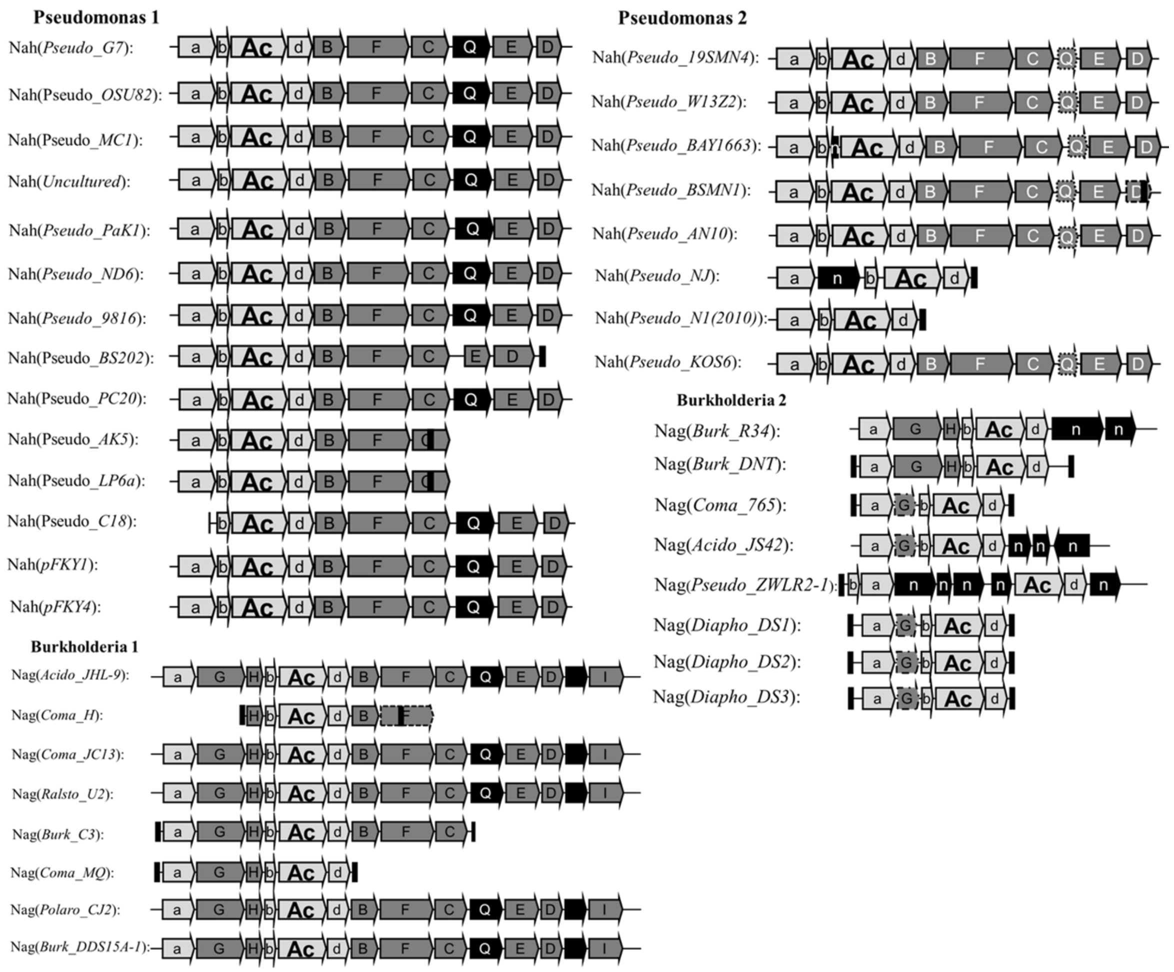

Fig. S7 Gene organization in *nah*-like gene clusters. Boxes in gray or deep gray pointing in the direction of transcription represent genes of PAHs degradation. For clarity of presentation, nahAaAbAcAd are shown as a,b,Ac,d, etc. Boxes in black are hypothetical proteins. Boxes with dashed borders are interrupted genes, incomplete sequences are indicated with short vertical bars on the side.

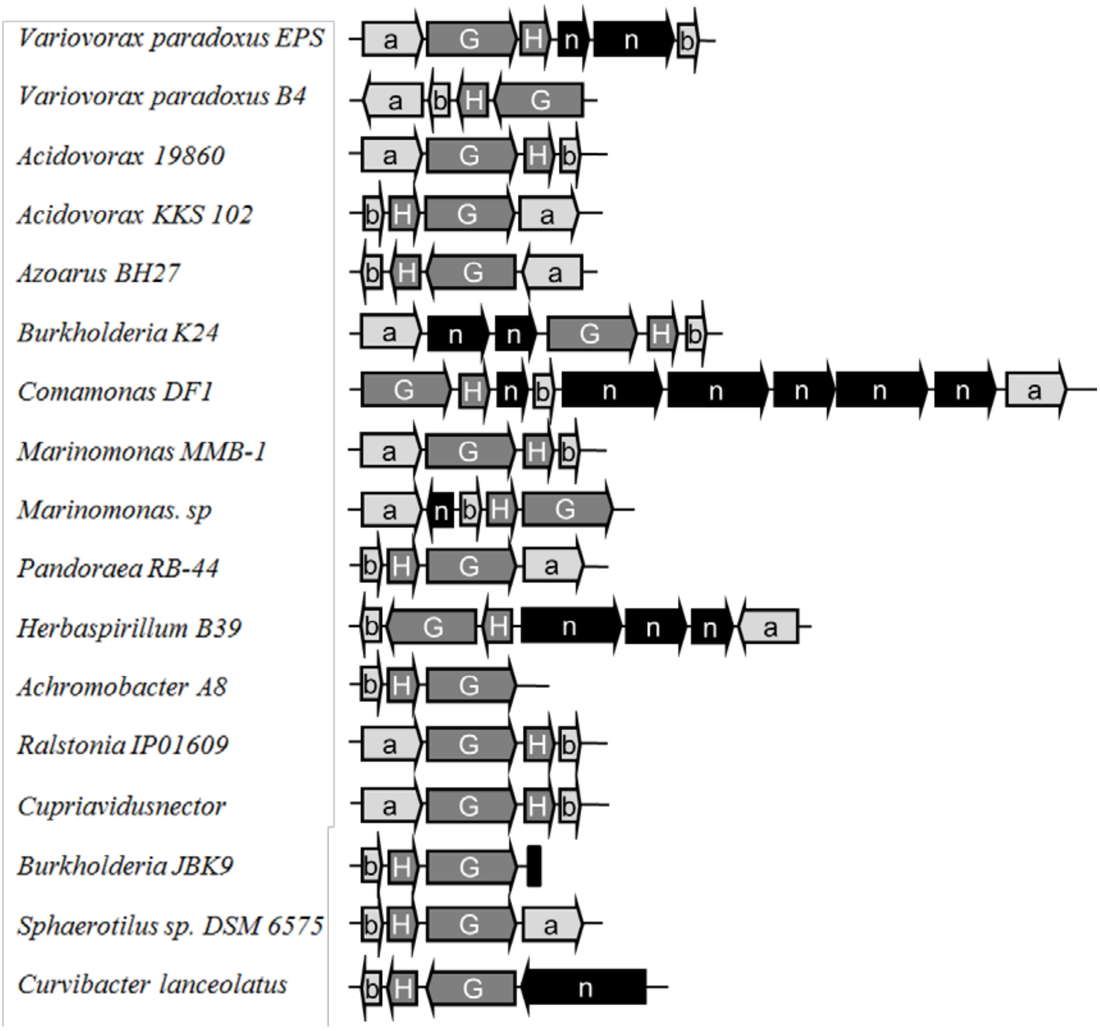

Fig. S8 Gene organization in Sal5H gene clusters. Boxes in gray or deep gray pointing in the direction of transcription represent Sal5H genes. For clarity of presentation, Sal5HAaAbGH are shown as a,b,G,H, etc. Boxes in black are hypothetical proteins.
